# Recommendations for delivering oral health advice: a qualitative supplementary analysis of dental teams, parents’ and children’s experiences

**DOI:** 10.1186/s12903-021-01560-w

**Published:** 2021-04-26

**Authors:** Amrit Bhatti, Karen Vinall-Collier, Raginie Duara, Jenny Owen, Kara A. Gray-Burrows, Peter F. Day

**Affiliations:** 1 School of Dentistry, University of Leeds, Clarendon Way, Leeds, LS2 9JT UK; 2 Bradford Community Dental Service, Bradford District Care NHS Trust, Bradford, UK

## Abstract

**Background:**

Tooth decay has a significant impact on children, their families and wider society. The dental consultation provides an opportunity to prevent tooth decay by engaging in an effective oral health conversation with parents and children. However, there is limited literature which explores how these oral health conversations are delivered, received, and understood.

**Aim:**

To explore the common facilitators of delivering oral health advice from dental teams, parents' and children's experiences, to identify and inform practical recommendations for clinical practice.

**Method:**

The current paper used a qualitative supplementary analysis to reanalyse data of existing published studies by applying a different research question. Qualitative focus groups were undertaken following a semi-structured interview guide with 27 dental team members (dentists, dental nurses, practice managers and receptionists), 37 parents and 120 children (aged 7–10 years old) in the northern region of England. Thematic analysis informed the identification of themes and aggregation of findings.

**Results:**

Three overarching themes were developed: (1) An engaging and personalised dental visit for parents and children; (2) Dental teams, parents and children working collaboratively to improve oral health habits; and (3) Recommending appropriate oral health products. Many parents and children had little recollection of any preventive oral health conversations when visiting the dentist. Practical solutions were identified by different stakeholders to facilitate three-way, personalised, non-judgemental and supportive oral health conversations. Adopting these innovative approaches will help to enable parents and their children to adopt and maintain appropriate oral health behaviours.

**Conclusion:**

Understanding the context and triangulating the experiences of stakeholders involved in preventive oral health conversations for young children is an essential step in co-designing a complex oral health intervention. This study has provided recommendations for dental practices and wider paediatric health care services. Furthermore, the findings have informed the design of a complex oral health intervention called "Strong Teeth".

**Supplementary Information:**

The online version contains supplementary material available at 10.1186/s12903-021-01560-w.

## Background

Tooth decay is common amongst children [1], especially in areas of deprivation [2]. From both a societal and health care perspective, tooth decay is a global problem [3] with wide-ranging negative effects on children [4, 5], their family [6, 7] and society [1]. Tooth decay, however, is preventable [8], with appropriate oral health behaviours established in the home-setting in early-childhood providing lifelong protective effects [9, 10]. These evidence-based oral health behaviours for young children include twice daily parental supervised toothbrushing with fluoride toothpaste and limiting sugary foods and drinks [11]. The term “optimal oral health habits” will be used though out this paper to describe these oral health behaviours. Parents and local communities identify their preference for establishing optimal oral health habits from the outset rather than correcting poor habits at a later stage [12]. To facilitate these optimal oral health habits, preventive programmes need to be multi-faceted and provide consistent oral health messages across all professionals involved in early-years care [13]. One key opportunity to provide oral health advice and guidance is when parents bring their child to the dentist.

Dental Check by One (an English initiative, which aims to promote dental attendance before a child’s first birthday) raises awareness of the opportunity for parents to take their child to the dentist in infancy. Enhancing access to dental services initiatives is common across the world and are endorsed by numerous professional bodies, including the European Academy of Paediatric Dentistry [14], American Academy of Pediatric Dentists [15], and the World Dental Federation [16]. National guidance (i.e., the “Delivering Better Oral Health" toolkit, Public Health England, 2017) provides advice on what information should be given to parents. However, evidence on how best to support parents adopt optimal oral health habits for their children at home is limited [17, 18] and is a key research priority [17].

To address this research gap, the first step was to understand the context and explore how oral health conversations were currently delivered, received and understood by dental team members, parents and young children. Our work (see Additional file 1: Table S3 for more details of these studies by Duara et al. [19–21]) involved undertaking focus groups with dental teams, parents and children separately to explore their individual experiences of dental consultations. Of equal importance, is the need to explore and synthesise the experiences of each individual groups and how they interrelate to each other. Indeed, there is a dearth of studies that have looked at the experiences and commonalities of this tripartite relationship. This current paper used a qualitative supplementary analysis [22] to reanalyse the original transcripts of the Duara et al. [19–21] studies with a different research question, as outlined in Table 1. Supplementary analysis, following Heaton [23], can be used to explore new or additional research questions upon data that were independently collected. This differs from other types of secondary analysis in that supplementary analysis allows the researchers to undertake a more in-depth analysis of an emergent issue, that were partially identified in the primary studies. This being, to triangulate the shared experiences of all the participants involved in delivering and receiving oral health advice for young children (i.e., dental team, parents and children).

**Table 1:** Summary of the different research questions for the current supplementary analysis versus the primary studies (Duara et al. 20–22)

Research Questions	
Primary studies [19-21]	Qualitative Supplementary Analysis (current paper)
(a) What are dental team members’ experiences of delivering oral health advice to children and their parents and caregivers?	
(b) What are the parent's experiences of receiving oral health advice from dental health professionals?	
(c) What are children’s experiences of receiving oral health advice from dental health professionals?	What are the experiences of dental teams, parents, and children related to the delivery of oral health advice?
What are the factors affecting parent’s oral health practices for their children?	What are the recommendations for dental teams to improve the delivery of oral health advice to young children's parents?
What do children know about oral health and what oral health behaviours do they perform?	

## Aim

To explore the common facilitators of delivering oral health advice from dental teams’, parents’ and children’s experiences to identify and inform practical recommendations for clinical practice.

## Methods

### Design

Analysis followed a qualitative descriptive approach using Thematic Analysis [24] at a semantic level which identifies the explicit and surface meanings of the data. AB re-examined the original studies by listening to the audio recordings, reading the transcripts and reading the original reports [19–21]. This qualitative supplementary analysis focused on new research questions, which expanded on the original study to identify commonly shared facilitators of delivering oral health advice to inform clinical practice and identify practical recommendations. As such, AB analysed the existing data based on this new research aim.

It is important to note that the primary researcher (AB) was not involved in the original focus groups and joined the research team after the studies had taken place. AB was a qualitative female research assistant who was familiar with using qualitative methods (PhD). AB reviewed the original documents, including transcripts, memos and notes. The researcher also had access to members of the original investigative team (KV-C, PD, JO, RD), to receive ongoing clarification.

### Sample

#### Dental team members

Dental team members were purposively sampled across Yorkshire and Lancashire (England) for their reputation of having a strong preventive ethos. In total, 27 dental team members, including dentists, dental nurses, practice managers and receptionists working within the NHS, corporate and private settings, took part in four focus groups. Some participants in the focus group worked within the same practice. Focus groups took place within practices and at a British Dental Association meeting.

#### Parents

Parents were purposively sampled across Yorkshire (England), including those living within the outskirts of Bradford, Leeds and Huddersfield. Parents and children were purposefully sampled within Bradford and the surrounding areas, given that the highest rates of childhood tooth decay is in Yorkshire (England), with almost 40% of children aged 5 years old having evidence of tooth decay compared to the national average of 23% [25]. In total, 37 parents took part, three as individual interviews and the rest as four focus groups within children’s centres, nurseries and primary schools settings. Individual interviews were undertaken at home to accommodate parents who had prior commitments but still wanted to take part in the study.

### Children

Six classes of primary school children took part in focus groups within school, with a total of 120 children aged 7–10 years old (purposive). Within each class, children were divided into groups of 8–10 children per researcher. Most of the schools were located just outside of Bradford city centre (England), except one, which was in a rural area outside of Huddersfield (See Additional File 1: Table S3 for further details of these focus groups). It is also important to note that during these focus groups, products, such as electric toothbrushes and toothpaste were used as visual aids/prompts to facilitate discussions.

Focus groups were undertaken by members of the research team (See additional file Table 1 for more detailed information and credentials).

### Ethical approval

Ethical approval was obtained by the Dental Research Ethics Committee (DREC), University of Leeds. Ref: 300317/PD/225.

### Analysis

AB, in collaboration with KV-C, primarily undertook the analysis of this data set with a new research question in mind. Following the process recommended by Braun and Clarke (2006), which entailed: (1) familiarisation with the data; (2) generating codes relevant to the aims of the study, and collating all the codes across dental team members, parents and children; (3) generating themes and sub-themes; (4) reviewing the themes; and (5) defining and naming themes. Data was organised, analysed and managed using NVivo.

Themes were developed by one researcher (AB), which captured the commonalities across dental team members, parents and children’s experiences that related to the facilitators of delivering and receiving oral health advice. Development of the themes was discussed with wider members of the research team (PD, KV-C, KG-B). It was an iterative process in which themes were developed and changed over time, owing to the nature of Thematic Analysis [24]. Throughout the process, multiple researchers (PD, AB, KG-B, KV-C, JO, RD) from different disciplines (Dentistry, Dental Public Health and Psychology) were involved in peer debriefing. The participants involved in the original studies did not provide feedback on the findings.

## Results

Three overarching themes were developed: (1) An engaging and personalised dental visit for parents and children; (2) Dental teams, parents and children working collaboratively to improve oral health habits, (3) Recommending appropriate oral health products.

### Theme 1: An engaging and personalised dental visit for parents and children

For parents and children, there appeared to be little recollection of what was discussed within the dental visit:

At the time they told me what fluoride content to look out for but I can’t remember now. (parent).

Different approaches (outlined below) from parents, dental teams and children, were suggested. Preference was shown for dental visits to be friendly, fun and engaging, but still providing key support and education. As such, the current theme identified ways in which providing an engaging dental visit may encourage recollection and how having an attractive environment, including posters and the use of technology (e.g., TV and digital screens) could make going to the dentist less intimidating and more welcoming.

#### Visual displays and resources engage parents and children

Many dental team members found that visual resources, such as posters within the clinic, helped draw the attention of both children and their parents. Utilising the dental waiting room for displays about oral health was seen as an efficient way to communicate oral health messages and stimulate conversations with the wider dental team, such as receptionists:

So at the moment we are doing the sugar display, we did the dummies, the juice in bottles on display. The sugar display... nearly every patient makes a comment about it. It’s really, really good and obviously hear any patients discuss it with their children, so if we hear any patients doing that we like to get involved. So I sort of listen and if you hear them talking you will sort of say and explain why we’ve done it. (Receptionist)

These findings reinforce how oral health can be delivered by the wider dental team, optimising every contact they have with the patient from the onset of the visit, including receptionists within the waiting area, making the delivery of oral health conversations more memorable. Within these dental practices, there appeared to be a whole team approach, which was not dependent on one staff member delivering oral health advice. An active engagement as shown by this receptionist was a positive way of delivering oral health messages to families of young children which enabled them to think about their oral health before they went into the clinic and spoke in more depth with the dentist.

#### Wanting a friendly and interesting environment

Dental team members, parents and children alike, described how attractive resources and technology are more likely to capture their attention. A relaxing and friendly environment could help with the enhanced delivery of oral health conversations:

like it could be... the walls could be more colourful and they could explain it more nicely because when I go to the dentist, they always shout at me [...] like they just say in a strict way like ‘you have [stresses the word] to...’. I want it to be more like nice, like more giggly. (Year 4 participant)

There was a desire for fun elements to be incorporated into their dental visit by using colourful displays, activities and rewarding their engagement with stickers, while also being informative. Having an environment that was appropriate to all audiences (i.e., child and adult) also appeared to be important. This is because some resources, such as leaflets, did not appeal to younger children; however, parents were more likely to engage with these and take them home. Interestingly, the child within the narrative above describes how the dentist appears to “tell off” the child and directly impose oral health messages. This, in turn, could make the dental visit appear intimidating for children and make it less likely that they will implement the advice given.

#### Involving the child within the dental visit

All participants identified the importance of involving children (aged 7–10 years old) in the visit. Dental visits provide opportunities for dental team members to talk to the child, as well as the parent, to help make them aware of the negative consequences of poor oral health:

Rather than speak to the parent, speak to them because obviously some kids, especially at school, when you’re talking to them direct, they listen more so one to one sessions are better […] (Nurse)

Children above the age of seven transition from being dependent on parents brushing their teeth, to exerting their own control and taking more responsibility for their oral health habits [11]. This, in turn, allows parents to take a more supervisory and motivational role in their child’s toothbrushing. The focus groups highlighted how the child could be actively involved when the advice is communicated:

If they notice a build-up of plaque anywhere then they will say you know you should be focussed on these areas. I would say my older boy has had x-rays and things recently and the dentist really had a good talk to us with that and like look at the x-ray and got him really quite involved with it which was really nice for him. (Parent)

They also said, ‘Do you use mouthwash?’. I said yes and they said, ‘Do you use after you brush your teeth’, I said ‘yes’ and they said to me, ‘Don’t really do it. If you brush your teeth in the morning, then come back from school and do it, not after you’ve brushed your teeth (Year 3 participant)

For children, how the message was conveyed was an important motivator, with oral health behaviours seen as advice *entrusted* to them rather than being *imposed* on them. This is important as dental team members can be perceived as telling patients what to do rather than exploring opportunities with patients, and thus these conversations were less likely to lead to behaviour change. The dental team members within the narrative of the current study had the opportunity to grasp the interest of the child by involving them within the oral health discussions. Having practical demonstrations (e.g., using disclosing tablets) alongside oral advice may have helped strengthen this engagement and recollection. Children within the focus groups showed an interest in being involved in these conversations and enjoyed viewing their x-rays and disclosed plaque. The dental team member in these instances had the potential to show why oral health behaviours were important, motivating children to place increased effort into maintaining optimal oral health habits.

### Theme 2: Dental teams, parents and children working collaboratively to improve oral health habits

Children were aware of the importance of optimal oral health and wanted the responsibility to look after their teeth. The narratives highlight the issues children and parents face undertaking oral health behaviours and the critical role parents play in maintaining optimal oral health habits.

#### Reminders for the child to brush

Parents within the focus groups saw morning toothbrushing as a part of their school routine. The evening brush, however, was often left to the child and therefore more vulnerable to being forgotten:

P: They said I have to brush in the morning and at night

I: Which one were you forgetting?

P: The night

I: Did they give you anything to remember to brush at night?

P: They said, ‘Your mum’s going to remind you at night’. (Year 4 participant)

Getting ready in the mornings were reported to be "hectic"; however, they appeared to be more organised because parents took control to ensure toothbrushing was done within the routine of getting ready for school. Evenings, however, were less time-pressured and structured, with children often responsible for getting themselves ready for bed. The narrative highlights the challenges of achieving regular bedtime brushing and how important it is for dental teams to explore these routines as they may help to identify opportunities to support good bedtime habits.

#### Supporting healthy eating and drinking habits

Although children felt responsible for controlling their eating and drinking habits, most spoke about how difficult it was for them to maintain a healthy diet, especially once unhealthy habits had been established:

I: Do you actually follow all the diet advice?

P1: Sometimes

P2: It’s a little hard

P3: Because it’s really hard to get out of it (Year 5 participants)

Interestingly, the narrative suggests that children, similar to adults, struggle to make healthy food choices, despite knowing what these are. This has been supported by dental team members who similarly discussed children’s regular access to sugary foods and drinks:

He was brushing his teeth but … with all this fizzy drinks, he was my first child so, I just let him loose! (Parent)

you will get some parents that are not interested. They’ve kicked off when I’ve said about the juices and they’ve said, ‘oh well if I don’t give them juice they’re gonna have a paddy’... and I’ve actually turned round and said, ‘look who is the parent here, this child does not go to a supermarket and buy the juice, it’s you that does it (Practice manager)

Although there is a shift in dependency, ultimately, it is the parent that has a crucial role in their child's oral health behaviours. Dental team members felt as though parents might have overlooked their role in regulating what food items are available to their children. Often, frustration was shown over who maintained responsibly in controlling sugary foods and drinks. Parents felt pressured to give in to children's demands as refusal could lead to uncooperative behaviour from the child, as shown by the phrase “going to have a paddy”.

Furthermore, the focus groups highlighted that parental attitudes significantly affected their children’s oral health behaviours. For example, a practice manager shared her experience with a patient's parents who refused to restrict sugary food because they were not convinced that it had severe repercussions on their child’s oral health:

but there are a lot of parents that take on what you say and some parents that say, ‘well I ate loads of sweets and it didn’t have any harm’. (Practice manager)

The dental team members reported difficulty in supporting parents who hold such strongly ingrained beliefs and were therefore more hesitant to deliver oral health advice. This narrative demonstrates how some parents may appear to be defensive and may normalise their child’s behavioural habits, forming a barrier towards dental team members as they perceive the advice to be a negative judgement.

#### Communicating optimal oral health messages to wider family and friends

Parents reported that social factors, such as school and cultural factors at home, strongly influenced their child’s sugar intake and lack of toothbrushing. The current sub-theme demonstrated the importance of communicating optimal oral health messages to wider family members and friends.

Some parents, for example, felt their partners were not as supportive in maintaining optimal oral health for their children:

Saying that, going to the dentist hasn't but now he's getting older he's getting lazy and it is a push to get him to do them. He will do them but, say if I'm at work and he's at home with his Dad, guaranteed he won't do his teeth (Parent)

While some parents felt their child was responsible for their oral health habits, others described how their partner or other family members did not share the same beliefs in the importance of maintaining optimal oral health habits. Changing these family norms was viewed as challenging, particularly when parents were perceived as unmotivated. Parents often struggled to relay oral health messages to other family members who cared for their child, especially when often only one parent attended the dental visits.

Parents highlighted a need for other care environments to be aware of, and enforce appropriate dietary behaviours, such as schools (e.g., not provide sweet snacks after lunch), specifically as the child grows older and spends more time away from the parent:

...it does concern me at school because we restrict sugary snacks at home but school doesn’t and I have actually written to local council about this. They offer at lunch time like puddings and cakes as well as fruit as an alternative but we all know what the children are going to go for so it’s kind of a bit deflating that we restrict but he isn’t obviously restricted at school. (Parent)

The narratives show the importance of consistent messaging for families and schools, who are integrally involved in children’s lives. It identifies the challenges of communicating with wider family members who may have significant responsibility for looking after children, but have not attended the dental visit.

### Theme 3: Recommending Appropriate Oral Health Products

The focus group discussions illuminated how recommendations of the appropriate oral health products can improve oral health behaviours, including toothbrushing for the right amount of time, motivating the child to brush, and establishing a good routine.

#### A focus on the practicalities of products

Dental team members discussed which dental care products they advised parents to use (e.g., toothbrushes and toothpaste), and were mindful of what products to recommend based on their price and long-term durability. Despite the preferences for, and the many advantages of, using electric toothbrushes, the cost of electric toothbrushes was viewed as a concern by dental team members:

I think for me cost is something that you have to factor into it because if you say to parents, ‘right you’ve got to buy an electric toothbrush and this toothpaste’, there’s no point as they don’t have that disposable income. So you’ve got to be realistic. So I always tell them about the food colouring and I always make sure they are not allergic to it first and say this is what we use here but if you want a cheaper alternative! (Practice manager)

Yeah, yeah I mean we got the Star Wars flashing Lightsaber one that kind of gives you a time limit of 2 minutes. It flashes for 2 minutes and makes noises for 2 minutes so yes that worked until it broke but it’s very expensive so we didn’t get it again but it did make him respond. (Parent)

Alternative products were suggested to parents by dental team members to increase the likelihood that they would follow and implement the oral health advice provided. This helped reduce barriers, by using a range of product costings and offering different options, such as food colouring rather than disclosing solutions or replacing the head of the electric toothbrush to allow other children to use. Within the narrative above, the child was motivated by the power toothbrush, and the inbuilt timer allowed him to brush for the allocated time.

Interestingly, many children and parents within the focus groups owned an electric toothbrush, indicating that assumptions made by the dental team regarding cost did not coincide with reality. The narrative highlights that parents are willing to buy appealing products, such as electric brushes if they were motivated to do so and could see the benefits of their purchase.

#### The importance of the products being attractive

The narratives highlight that children were often responsible for choosing which toothbrush to buy, and displayed a preference for electric toothbrushes, initially for their aesthetics and later for their practicality. This availability of attractive toothbrushes could potentially increase children to undertake optimal oral health behaviours:

I: Who chooses the toothbrush for you and what do you prefer?

P1: I choose my toothbrush. I normally choose the one that is electric [...] I would choose that one [pointing to a product displayed] because it is FROZEN (Year 3 participant)

The characters often grabbed children’s attention and increased the chance that they would ask their parents to buy a specific toothbrush:

If I was younger and I liked CARS and things, I would brush with that. It might make me like it more because it has CARS. (Year 5 participant)

As children grow older, however, the novelty of these characters could fade, and popular children's characters may be less likely to influence their choice. This was shown by Year 5 children who focused on the ease of electronic toothbrushes rather than the characters displayed:

I: Why do you prefer the electric toothbrush?

P1: It’s easier

P2: Less energy needed

P3: It cleans your teeth better [...] and the round ones are better (Year 5 participants)

Children often favoured the electric toothbrush because they believed it was easier to use and more convenient compared to a manual toothbrush.

#### The difficulties of transitioning onto stronger tasting fluoride toothpaste with higher fluoride content

Although children usually chose their toothbrushes, the focus groups identified that parents decided which toothpaste to buy. National guidance recommends that children should transition to toothpaste containing between 1350 and 1500 parts per million (ppm) fluoride around six years old or earlier if the child is at high risk of tooth decay [11]. Some parents, however, reported the difficulty in transitioning their children from flavoured infant toothpaste (of around 1000 ppm and usually sweet or mildly mint flavoured) to a child toothpaste (of up to 1500 ppm fluoride) due to the strong mint flavour.

I think they once had a go with ours but they found it too strong so, I bought a child’s […] I’ve never read it to be honest. (Parent)

Following on from the notion that parents and children struggle to remember the advice given within the dental visit, including the appropriate fluoride content for their child (theme one), the narrative above suggests that parents may therefore look for toothpastes that are targeted towards infants or children, which may not match the correct fluoride content for their age. This could, however, cause longer term problems because it is more difficult to migrate to higher strength toothpastes with stronger mint flavours which are more appropriate:

I’m always an advocate for not using fruity flavoured toothpaste. Try and get them on mint because as soon as they’re too old for the fruit stuff, it’s a shock to the system and they stop brushing their teeth because they don’t like it. (Dental Nurse)

Therefore, some dental team members recommended using a small amount of family toothpaste with the stronger fluoride content from the outset to desensitise children to the strong taste and prevent later transitioning difficulties.

## Discussion

This study has examined the shared experiences of dental team members, parents, and children within the dental setting. Findings have identified the needs of those involved when undertaking oral health behaviour change conversations with parents of young children. Specifically, making the dental visit engaging and personalised; a collaborative effort between dental team members, parents, and children to improve oral health habits, and dental team members recommending the appropriate oral health products. Identifying commonalities of this tripartite relationship is key to improving the quality and delivery of oral health conversations, particularly to those who may appear resistant.

The long-term plan for the NHS (the National Health Service) places a clear emphasis on primary prevention [26]. While dental teams undertake preventive conversations at the dental consultation (as identified in this research) a majority of children and parents had little recollection of these conversations. This reinforces the pressing need for effective interventions in the dental setting which empower children and their families to embrace self-care and establish appropriate oral health habits. As outlined by the Medical Research Council's guidance [27] for complex interventions, this research is the critical first step to understand the experiences and context of those involved. Through supplementary analysis, the researchers synthesised the experiences of dental team members, parents and children and highlighted their complex interactions. The research informed the co-design of a complex intervention called “Strong Teeth”, with key findings and recommendations shown in Table 2.

**Table 2:** Themes and sub-themes of common facilitators to behaviour change conversations amongst dental team members, parents and children, and recommendations for practice

Themes	Sub-themes	Description of sub-themes and recommendations for practice
An engaging and personalised dental visit for parents and children	Visual displays and resources to engage parents and children	Visual resources, such as posters within the clinic, helped draw the attention of children and their parents. Displays and resources (e.g., both digital and paper-based) were viewed as an effective method to initiate oral health conversations that can be continued by different members of the dental team throughout the dental visit
	Wanting a friendly and interesting environment	Participants shared a preference for the dental clinic to be attractive and engaging to encourage conversations that are remembered by parents and children
	Involving the child within the dental visit	Discussions with parents and children highlighted that advice was usually directed at the parent rather than the child. There is a balance between empowering the older child to engage with their oral health behaviours while reinforcing the importance of parents providing oversight
Dental teams, parents and children working collaboratively to improve oral health habits	Reminders to brush	While the morning brush was often completed and ingrained into their routine, children described how evening brushing was often forgotten. Exploring how morning and evening toothbrushing behaviours differ and examining the barriers and facilitators to each allowed personalised non-judgement conversations to take place
	Supporting healthy eating and drinking habits	Children spoke about how difficult it was for them to refrain from consuming sugary items even though they know they were not good for them. Dental team members felt that parents needed to be aware of their critical role in supporting their child’s healthy eating habits and in managing their child’s wider environment
	Communicating optimal oral health messages to wider family and friends	Communicating optimal oral health messages to wider family and friends, such as limiting sugary snacks and the importance of toothbrushing was described as difficult for both parents and dental team members. Exploring these challenges can allow dental team members to develop a rapport with parents and open up this important topic of conversation
Recommending appropriate oral health products	A focus on the practicalities of products	Dental teams were mindful of which products to recommend based on the price and long-term use of the products. However, many parents and children reported owning such products. Dental teams can help by providing appropriate advice on suitable products where parents are keen to find out more
	The products should be attractive	Children were often responsible for choosing which toothbrush to buy. Therefore, products that were attractive to them were more likely to be bought and can motivate children to engage with optimal oral health behaviours
	The difficulties of transitioning onto stronger tasting fluoride toothpaste	Parents frequently purchase their child’s toothpaste without children having a role in the choice of what to buy. The stronger mint flavour of adult/family toothpaste can act as a barrier to transitioning from a child to adult toothpaste as the permanent teeth erupt. This caused difficulty for both parents and dental team members who want to encourage the transition to a toothpaste with higher fluoride content. Recommending a very small amount of adult/family toothpaste from the outset with careful application by an adult can prevent these later difficulties

The study has highlighted the importance of an engaging and personalised dental visit. The literature [21, 28, 29] reports that many oral health conversations are one-way conversations with a didactic delivery and involve a simple transfer of information. The variance of parent and child recollections from these conversations demonstrate the limitation of such approaches. While a “fun and engaging” visit may not on its own lead to behaviour change, when combined with other communication skills, they can help to develop rapport, active listening, and reciprocal and non-judgemental conversations [30]. Such collaborative approaches allow parents and children to identify small specific steps to improve current habits as well as motivating them to engage with these new behaviours.

Earlier studies have identified how a whole team approach is essential to reduce oral health inequalities and requires the active engagement of all members of the dental team [31]. This is consistent with the established policy guidelines of ‘Making Every Contact Count’ (MECC) NHS Health Education England [32] which emphases the important role all health professionals play by taking all opportunities for behaviour change conversations to support the adoption of healthier behaviours. The benefits, acceptability and financial remunerations of using the wider dental team in this role is rapidly evolving within the UK [29, 33]. There is a particular challenge on how to best address and engage children of different ages, especially as children become more independent and take responsibility for some oral health behaviours. Although this may be regarded as self-evident, ways of exploring how to make this three-way conversation work effectively have received little attention. Having visual displays and resources, as well as providing a friendly and interesting environment, can help engage parents and children and make the advice more memorable. Such findings not only apply within the dental practice but also wider paediatric health services that can utilise the wider team and enhance the opportunities to undertake effective conversations with parents of young children at every contact. In addition, it raises the question of how dental teams can engage at wider family and community levels to enable a “whole system” approach, across early-years services and embed optimal oral health behaviours as a social norm.

The findings from the current study have also identified that children (aged 7–10 years old) appeared to play a more significant role in their toothbrushing and healthy eating habits than anticipated by dental team members. This suggests there is a balance between encouraging children to take responsibility for their own oral health habits while empowering parents to maintain active engagement in their child’s oral health behaviours. These findings have been similarly expressed in the wider literature [34–36], where parents shifted their role of implementing optimal oral health habits to the child or the wider family. The theme describes how some parents can appear resistant to take on this difficult supervisory (and where necessary, enforcement) role. Dental teams often identified these resistant parental attitudes as one of the most challenging and demotivating experiences, suggesting a need for further training in communication skills and use of specific techniques such as motivational interviewing and rolling with resistance [34]. The wide variation in children’s daily oral health routines described by parents and children reinforces the need for dental teams to explore home practices to enable personalised and non-judgement supportive guidance to be provided.

### Strengths and limitations of the study

This study uses a supplementary analysis of existing qualitative data to triangulate the findings of how oral health conversations are delivered and received within a dental practice. The synthesis of parents, children and dental team members experiences within one study is a critical step in designing oral health interventions that are suitable to all those who are involved. Being mindful of different perspectives from stakeholders is an essential component of clinically successful interventions [37], and NICE guidance has highlighted the limited rigour within intervention development [38]. Often interventions overlook the context and the background during their development [39]. The current study also illuminates the views of children within research as equal to parents and dental teams. There appears to be a dearth of research that has focused on this triadic interaction and, in particular, the impact of dental team members communication on children and their parents [40]. Instead, studies appear to explore the dyadic interactions between doctors/dentists and their adult patients [41]. Existing health research has traditionally been conducted *on* children, whereas the current study has research*ed with* children [42].

The supplementary analysis has been applied to consolidate the commonalities between dental teams, parents and children. This has deepened our understanding of the experiences of oral health care advice and factors, which influence oral health practices. While the use of this approach can be considered a strength of this study, the potential limitations of this should also be considered. Firstly, the study was a broadly inductive approach in which the analysis involved prior research questions and the aims of the primary study directed how the questions were presented to the participants. As such, scholars have criticised supplementary analysis for not encouraging divergent thinking and limiting the scope of analysis [43]. In line with the nature of secondary analysis [43], the questions posed within the original data were not specifically for the purpose of this paper. As such, specific probing questions relating to facilitators of optimal oral health care advice may have elicited different responses from participants. In addition, the scope of the current paper was to explore commonalties across the participant groups and therefore a negative case analysis was not undertaken. This may have highlighted divergent cases which were not presented. However, the inclusion of multiple researchers in the analysis phase to compare interpretations of the data, facilitated data verification and ensured that the data aligned to the aims of the study. Finally, given that this study was conducted in the North of England, the findings presented in this paper should be cautiously generalised to other countries due to cross-cultural differences and access to dental services worldwide.

Although the products were used within the original focus groups as prompts to facilitate discussions, responses may have been guided by the specific products rather than an overall view of the whole range of products available. However, the studies encouraged all participants to share their views openly and honestly. Furthermore, some participants may have responded in a socially desirable way, especially to match that of their peers. Generalisability should also be cautioned. Dental team members were recruited because of their reputation for having a strong preventive ethos; thus, the experiences of these may differ from "regular" practices. Nonetheless, the practices recruited (e.g., those working across the NHS and private dental care and with different ownership models, including both corporate and small partnership-based practices) should encourage a diversity of views.

## Conclusion

This study is an essential step in providing the context and understanding of preventive oral health conversations within the dental setting. Through triangulating experiences, specific examples of good practice have been identified with potential utility for wider paediatric health care services. Resistance to changing oral health behaviours and managing these conversations has identified the need for training and support around specific behaviour change techniques. In particular, how to have a personalised non-judgemental two-way conversation, rolling with resistance and understanding the wider context of those involved in the child's daily routine. The findings of this study have informed the co-design of a complex preventive oral health intervention, called “Strong Teeth, " which aims to support optimal oral health habits for children through effective oral health conversations with the dental team.

## Supplementary Information

**Additional File 1.** **Table S3:** A table to summarise the studies by Duara et al. [19–21].

**Additional File 2.** Topic guides by Duara et al. [19–21].

## Data Availability

Further information of the data set and materials can be available from the corresponding author on reasonable request.

## Abbreviations

MRC: Medical Research Council
DREC: Dental Research Ethics Committee
NHS: The National Health Service
MECC: Making Every Contact Count
NICE: National Institute for Health and Care Excellence

## References

[1] Public Health England. Health Matters: Child dental health 2017 [Available from: https://publichealthmatters.blog.gov.uk/2017/06/14/health-matters-child-dental-health.

[2] Rouxel P, Chandola T (2018). Socioeconomic and ethnic inequalities in oral health among children and adolescents living in England, Wales and Northern Ireland. Commu Dent Oral Epidemiol.

[3] Tinanoff N, Baez RJ, Diaz Guillory C, Donly KJ, Feldens CA, McGrath C (2019). Early childhood caries epidemiology, aetiology, risk assessment, societal burden, management, education, and policy: global perspective. Int J Pediatr Dent.

[4] Ramos-Jorge J, Pordeus IA, Ramos-Jorge ML, Marques LS, Paiva SMJCd (2014). Impact of untreated dental caries on quality of life of preschool children: different stages and activity. Commu Dent Oral Epidemiol.

[5] Abanto J, Tsakos G, Paiva SM, Carvalho TS, Raggio DP, Bönecker M (2014). Impact of dental caries and trauma on quality of life among 5- to 6-year-old children: perceptions of parents and children. Commun Dent Oral Epidemiol.

[6] Martins-Júnior P, Vieira-Andrade R, Corrêa-Faria P, Oliveira-Ferreira F, Marques L, Ramos-Jorge M (2013). Impact of early childhood caries on the oral health-related quality of life of preschool children and their parents. Caries Res.

[7] Gomes MC, de Almeida Pinto-Sarmento TC, de Brito Costa EMM, Martins CC, Granville-Garcia AF, Paiva SMJH (2014). Impact of oral health conditions on the quality of life of preschool children and their families: a cross-sectional study. Health Qual Life Outcomes.

[8] Gilchrist F, Marshman Z, Deery C, Rodd HD (2015). The impact of dental caries on children and young people: what they have to say?. Int J Pediatr Dent.

[9] Broadbent JM, Thomson WM, Boyens JV, Poulton R (2011). Dental plaque and oral health during the first 32 years of life. J Am Dent Assoc.

[10] Hall-Scullin E, Whitehead H, Milsom K, Tickle M, Su T, Walsh T (2017). Longitudinal study of caries development from childhood to adolescence. J Dent Res.

[11] Public Health England. Delivering better oral health: an evidence-based toolkit for prevention England: Public Health England and Department of Health; 2017. Available from: https://assets.publishing.service.gov.uk/government/uploads/system/uploads/attachment_data/file/605266/Delivering_better_oral_health.pdf.

[12] Gray-Burrows K, Day P, Marshman Z, Aliakbari E, Prady S, McEachan R (2016). Using intervention mapping to develop a home-based parental-supervised toothbrushing intervention for young children. Implement Sci.

[13] Public Health England. Commissioning Better Oral Health. London: https://www.gov.uk/government/uploads/system/uploads/attachment_data/file/321503/CBOHMaindocumentJUNE2014.pdf; 2014.

[14] European Academy of Paediatric Dentistry. Guidelines on prevention of early childhood caries: an EAPD policy document. 2008. Available from: https://www.eapd.eu/uploads/1722F50D_file.pdf.

[15] American Academy of Pediatric Dentists. American Academy of Pediatric Dentistry (AAPD) 2018. Available from: https://www.aapd.org/about/about-aapd/who-is-aapd/.

[16] FDI World Dental Federation (2014). FDI policy statement on dental amalgam and the Minamata Convention on Mercury: adopted by the FDI General Assembly: 13 September 2014, New Delhi, India. Int Dent J.

[17] NICE. Oral health promotion: general dental practice 2015. Available from: https://www.nice.org.uk/guidance/ng30/chapter/Recommendations-for-research.

[18] James Lind Alliance. Priority 1 from the Oral and Dental Health PSP 2018. Available from: http://www.jla.nihr.ac.uk/priority-setting-partnerships/oral-and-dental-health/priority-1-from-the-oral-and-dental-health-psp.htm.

[19] Duara R, Vinall-Collier K, Owen J, Day P. Final Report to Funder- Dental professional’s experiences of delivering oral health advice to children and their parents/caregivers: Focus groups with dental practitioners and their wider teams. White Rose Research Online; 2019.

[20] Duara R, Vinall-Collier K, Owen J, Day P. Final Report to Funder- Parent’s experiences of receiving oral health advice from dental health professionals for their children and factors influencing oral health practices: Focus groups with parents of children aged 0–11 years. White Rose Research Online; 2019.

[21] Duara R, Vinall-Collier K, Owen J, Day P. Final Report to Funder- Children’s experiences of receiving oral health advice from dental professionals and oral health behaviours: Focus groups with children aged 7–10 years. White Rose Research Online; 2019.

[22] Heaton J (2004). What is secondary analysis.

[23] Heaton J. Secondary analysis of qualitative data: an overview. Hist Soc Res. 2008:33–45.

[24] Braun V, Clarke V (2006). Using thematic analysis in psychology. Qual Res Psychol.

[25] Public Health England. Oral health survey of 5 year old children 2017 England: Gov.uk; 2018 Available from: https://www.gov.uk/government/statistics/oral-health-survey-of-5-year-old-children-2017.

[26] NHS. The NHS long term plan. London, Health Do; 2019.

[27] MRC. Developing and evaluating complex interventions: new guidance. www.mrc.ac.uk/complexinterventionsguidance; 2006.

[28] Bhatti A, Vinall-Collier K, Duara R, Owen J, Gray-Burrows K, Day P. Dental Teams, Parents and Children’s Experiences of Oral Health Advice: A Supplementary Analysis. The Journal of Clinical and Translational Research. 2020. 10.1186/s12903-021-01560-w PMC8077708 33902541

[29] Pine C, Adair P, Burnside G, Brennan L, Sutton L, Edwards RT, et al. Dental RECUR Randomized Trial to Prevent Caries Recurrence in Children. Journal of Dental Research. 2020:0022034519886808. 10.1177/0022034519886808 31944893

[30] Kay E, Vascott D, Hocking A, Nield H, Dorr C, Barrett H (2016). A review of approaches for dental practice teams for promoting oral health. Commun Dent Oral Epidemiol.

[31] Watt R, Williams D, Sheiham A (2014). The role of the dental team in promoting health equity. Br Dent J.

[32] MECC. Making Every Contact Count 2012 [Available from: https://www.makingeverycontactcount.co.uk/.

[33] Galloway J, Gorham J, Lambert M, Richards D, Russell D, Russell I (2002). The professionals complementary to dentistry: systematic review and synthesis.

[34] Aljafari AK, Gallagher JE, Hosey MT (2015). Failure on all fronts: general dental practitioners' views on promoting oral health in high caries risk children–a qualitative study. BMC Oral Health.

[35] Watt RG (2012). Social determinants of oral health inequalities: implications for action. Commun Dent Oral Epidemiol.

[36] Ahmad A, Jennifer G, Marie H (2015). Failure on all fronts: general dental practitioners’ views on promoting oral health in high caries risk children-a qualitative study. BMC Oral Health.

[37] Peláez S, Bacon SL, Lacoste G, Lavoie KL (2016). How can adherence to asthma medication be enhanced? Triangulation of key asthma stakeholders' perspectives. J Asthma.

[38] NICE. Oral health: approaches for local authorities and their partners to improve the oral health of their communities. 2014.

[39] Aliakbari E, Gray-Burrows K, Vinall-Collier K, Edwebi S, marshman Z, Rosie.mceachan, et al. Home-based toothbrushing interventions for parents of young children to reduce dental caries: a systematic review. Int J Paediatr Dent. 2020. 10.1111/ipd.12658 32333706

[40] Yuan S, Humphris G, Ross A, MacPherson L, Zhou Y, Freeman R (2018). A mixed-methods feasibility study protocol to assess the communication behaviours within the dental health professional-parent-child triad in a general dental practice setting. Pilot Feasibility Stud.

[41] White RO, Eden S, Wallston KA, Kripalani S, Barto S, Shintani A (2015). Health communication, self-care, and treatment satisfaction among low-income diabetes patients in a public health setting. Patient Educ Couns.

[42] Marshman Z, Gupta E, Baker SR, Robinson PG, Owens J, Rodd HD (2015). Seen and heard: towards child participation in dental research. Int J Pediatr Dent.

[43] Clarke SP, Cossette S. Secondary analysis: Theoretical, methodological, and practical considerations. Can J Nurs Res Arch. 2016;32(3). 11928128

[44] Watt RG, Sheiham A (2012). Integrating the common risk factor approach into a social determinants framework. Commun Dent Oral Epidemiol.

[45] Watt RG (2012). Social determinants of oral health inequalities: implications for action. Commun Dent Oral Epidemiol.

[46] Witton RV, Moles DR (2013). Barriers and facilitators that influence the delivery of prevention guidance in health service dental practice: a questionnaire study of practising dentists in Southwest England. Commun Dent Health.

