# Supplementary material for: Recommendations for delivering oral health advice: a qualitative supplementary analysis of dental teams, parents’ and children’s experiences
Source: BMC Oral Health. 2021 Apr 26;21:210. doi: 10.1186/s12903-021-01560-w (PMC8077708; doi:10.1186/s12903-021-01560-w)
Supplement: Supplementary file 1 — Additional File 1. Table S3: A table to summarise the studies by Duara et al. [19–21]. [file 12903_2021_1560_MOESM1_ESM.docx]

Appendix 1: Table 3

A table to summarise the studies by Duara et al. (2019)

| Reference | Aim | Sample | Method | Analysis | Themes & Key Findings |
| --- | --- | --- | --- | --- | --- |
| Duara et al. (2019b)  Duara R, Vinall-Collier K, Owen J, Day P. 2019. Final report to funder- dental professional’s experiences of delivering oral health advice to children and their parents/caregivers: Focus groups with dental practitioners and their wider teams. White Rose Research Online. | To explore dental team members’ experiences of delivering oral health advice (e.g., toothbrushing and diet) to children and their parents and caregivers. | Dental team members including dentists, dental nurses, practice managers and receptionists  practicing in both the NHS and private settings.  Some participants within the focus group worked within the same practice.  Purposively sampled across Yorkshire and Lancashire (England) for their reputation of a strong preventive ethos  N=27 participants | Four focus groups.    Settings:  Dental practices (within Yorkshire) and British Dental Association meeting (Lancashire)  Data collection period: April- May 2017  Consent:  Information sheets were distributed to dental team members at least five days before the focus groups. Participants had to complete the consent form to participate.  Facilitated by two researchers:  RD & JO  Time taken for focus groups: 60 minutes  A topic guide (see appendix 2) and oral health resources, provided by P&G, were used to facilitate the conversations.  Focus groups were audio-recorded and professionally transcribed in verbatim. | Qualitative descriptive approach.  Thematic analysis carried out by one researcher (RD) in collaboration with other members of the research team.   1. Reading and re-reading of text to identify the main themes and patterns within the data. Individual or unique data were also noted down. 2. Re-read of text to find commonalities or contradictions. 3. Sub-themes and overarching themes were subsequently developed and refined by a discussion between the research team. All key themes were then reviewed. 4. Final themes were agreed and named and the report produced as a collaborative piece of work between members of the research team.   Negative case analysis was also undertaken. | 1. *Essential Teamwork*   Dental teams identified the importance of delivering preventive advice using a whole team approach.   1. *Passion and dedication*   An ideal attribute was to be dedicated and motivated to deliver oral health advice. This was especially because of the limited time, funding and resources within the dental setting.   1. *Communicating the message*   Dental teams felt it was important for the child to develop a sense of authority and responsibility when looking after their teeth. When parents were not aware of the serious implications of behaviours, dental team members would often focus on their guilt or emotion. The use of general visual displays in the reception area were seen as effective in grabbing attention and facilitating conversations.   1. *School – an ideal environment*   There were frustrations over the lack of support and funding to provide opportunities to engage with children within schools.   1. *Products – focus on the practicalities*   Dental teams felt they should be sensitive about the cost of oral health products. Preference was shared for adult toothpaste, rather than flavoured. |
| Duara et al. (2019c)  Duara R, Vinall-Collier K, Owen J, Day P. 2019b. Final report to funder- parent’s experiences of receiving oral health advice from dental health professionals for their children and factors influencing oral health practices: Focus groups with parents of children aged 0-11 years. White Rose Research Online. | To explore parent’s experiences of receiving oral health advice (e.g., toothbrushing and diet) from dental health professionals and the factors affecting the oral health practices of their child. | Parents were purposively selected across Yorkshire (England), including those living in or on the outskirts of Bradford, Leeds and Huddersfield.  Recruitment settings:  Children’s centres, nurseries and primary schools.  Parent’s socio-economic backgrounds and ethnicities varied.  N=37  Parents were purposively sampled based on the area in which they lived (within the top 30% of the most deprived). | Four focus groups & three individual interviews  Settings: Children’s centres, nurseries and primary schools.  Data collection period: April- May 2017  Consent:  Participant information sheets were distributed to parents at least five days before the focus groups or interviews. To participate, each parent had to complete the consent form.  Facilitated by three researchers:  RD, JO & KV-C  Time taken for focus groups: 50 minutes  A topic guide (see appendix 2) and oral health resources, provided by P&G, were used to facilitate the conversations.  Focus groups were audio-recorded and professionally transcribed in verbatim. | Qualitative descriptive approach.  Thematic analysis carried out by one researcher (RD) in collaboration with other members of the research team.   1. Reading and re-reading of text to identify the main themes and patterns within the data. Individual or unique data were also noted down. 2. Re-read of text to find commonalities or contradictions. 3. Sub-themes and overarching themes were subsequently developed and refined by a discussion between the research team. All key themes were then reviewed. 4. Final themes were agreed and named and the report produced as a collaborative work between members of the research team.   Negative case analysis was also undertaken. | 1. *Instruction and execution*   Parents stated that advice for their child during dental visits was limited. Other family members (e.g., partners) undermined parents’ attempts to care for their child’s oral health. They also reported difficulties in toothbrushing and dietary control as the child became older. Parents suggested a need for other care environments (e.g., school) to be aware of and enforce appropriate dietary behaviours.   1. *Direct communication with the child*   Parents suggested that the dentist could involve the child as well as the parent when communicating oral health advice. Furthermore, they felt that dental team members could emphasise the consequences of poor oral practices to the child by using visual methods. They also suggested delivery methods that would be more likely to engage children with advice, such as the use of technology (e.g., TV screens) rather than just informational leaflets.   1. *Awareness from other sources*   Preference was shared for oral health to be delivered within school and by health visitors.   1. *Product preferences*   Fictional characters on products were viewed as motivating for children. Parents took responsibility for choosing which toothpaste to buy. Some parents also reported the difficulty in transitioning their children from flavoured children’s toothpaste to an adult toothpaste. |
| Duara et al. (2019a).  Duara R, Vinall-Collier K, Owen J, Day P. 2019a. Final report to funder- children’s experiences of receiving oral health advice from dental professionals and oral health behaviours: Focus groups with children aged 7-10 years. White Rose Research Online. | To explore children’s experiences of receiving oral health advice from dental health professionals, and explore their oral health knowledge and behaviours (e.g., toothbrushing and diet). | Children aged 7-10 years old  Recruitment: Children from three different schools formed the sample population and were invited by a letter sent from the school to take part.  N=120  These schools were purposely sampled based on  the catchment areas. Children within schools 1 & 2 were based within the top 30% of the most deprived. Children within school 3 were based within the top 30% of the most affluent neighbourhoods in England .  Due to the time of year the focus groups were undertaken, children younger and older than the above ages were unable to participate as they were preparing for national exams. | Six focus groups: Two Year 3 classes (aged 7-8 years old), one Year 4 class (aged 8-9 years old) and two Year 5 classes (aged 9-10 years old).  Consent:  Information sheets were distributed parents at least five days before the focus groups. Parents had to complete the consent form for their child to participate.  Settings: School 1 and 2 were located just outside of Bradford city centre. School 3 was located in a rural area outside Huddersfield(England)  Facilitated by:  PD, KV-C, SW, JO, ABH, RD.  Dental education was also delivered by a paediatric dentist (PD).  Teachers of the children were present within these focus groups.  Data collection period:April- May 2017  Time taken for focus groups: 40-50 minutes. The students reconvened as a whole class and dental education was delivered by a paediatric dentist (PD).  A topic guide (see appendix 2) and oral health resources, provided by P&G, were used to facilitate the conversations.  Focus groups were audio-recorded and professionally transcribed in verbatim. | Qualitative descriptive approach.  Thematic analysis carried out by one researcher (RD) in collaboration with other members of the research team.   1. Careful reading and re-reading of text to identify the main themes and patterns within the data. Individual or unique data were also noted down. 2. Re-read of text to find commonalities or contradictions. 3. Sub-themes and overarching themes were subsequently developed and refined by a discussion between the research team. All key themes were then reviewed. 4. Final themes were agreed and named and the report produced as a collaborative work between members of the research team.   Negative case analysis was also undertaken. | 1. *Stimulating dental visits:*   When discussing their preferences for dentist visits, children wanted their dental visit to be stimulating and the dental setting to be an attractive environment. They also wanted their dentist to be friendly.   1. *Awareness and implementation*   Children were aware of brushing twice a day and limiting sugar consumption. However, children were unsure of when to brush (before or after breakfast) and indicated that the night time brush was more likely to be forgotten. It was difficult to refrain from sugary food. The findings suggested that the responsibility for looking after their oral health is more appropriate for older children who are able to use apps to monitor their sugar intake.   1. *Motivation to change*   Attractive oral care products (e.g., electric toothbrushes and phone apps) appeared to motivate children with developing good oral health habits as well as being aware of the negative consequences of poor oral health habits. Children were also interested in technology, such as electric toothbrushes and phone apps for toothbrushing and games.   1. *Oral health products*   Children generally preferred the electric toothbrushes compared to manual toothbrushes due to ease of use and functionality. |
