# Supplementary material for: Recommendations for delivering oral health advice: a qualitative supplementary analysis of dental teams, parents’ and children’s experiences
Source: BMC Oral Health. 2021 Apr 26;21:210. doi: 10.1186/s12903-021-01560-w (PMC8077708; doi:10.1186/s12903-021-01560-w)
Supplement: Supplementary file 2 — Additional File 2. Topic guides by Duara et al. [19–21]. [file 12903_2021_1560_MOESM2_ESM.docx]

Appendix 2- Topic guides from Original Studies

The dental teams perceptions of the barriers and facilitators to providing preventive oral health advice and support to children (0-11 years old) and their parents in a general dental practice setting- TOPIC GUIDE

**Current practices:**

Maybe we could start with some “warm up” questions - which patients do you normally see? Do you often visits kids ? which age groups ? any differences in between the age groups?

Please describe how you and your team in general dental practice delivers oral health advice and support to children (0-11 years old) and their parents?

Who delivers it?

What does the intervention look like?

Who does most of the talking? (dental team, parent, child)

How does the intervention change for children of different ages between 0-11 years old?

What areas of oral health advice does it concentrate on (toothbrushing, fluoride, diet, others)?

What guidance do you follow when giving oral health advice?

How do you explain the different oral care behaviors? Which tools do you use?

How long do you spend?

In what environment is it delivered (in surgery, in a specific oral health promotion room, other)?

What resources do you use? Does it include a “hands on” component e.g. brushing instruction?

What materials (and or rewards) do you give to children and their parents to take home?

Do you feel you have the necessary training, skills and experience to deliver oral health advice and support to this age group?

How do children and their parents respond to oral health advice and support?

Do you feel your oral health advice and support intervention is effective at supporting children and their parents to adopt appropriate oral health behaviours? Why?

**Facilitators:**

In the ideal world how would you design and deliver a preventive oral health advice and support intervention to children (0-11 years old) and their parents?

What would your intervention look like? – Do you know what it would look like?

How would you deliver it?

Who would deliver it?

Describe the environment it would be delivered in?

What resources would you use?

How would the intervention change for different age groups?

How would you address wider social and environment influences?

How would these resources help with delivering oral health advice and support?

What additional resources would help you with delivering oral health advice and support?

**Barriers:**

In the previous conversation, you described your ideal design of an intervention to encourage children (aged 0-11 years old) and their parents to adopt and maintain appropriate oral health behaviours – in the general practice where you work, what prevents you from delivering this ideal intervention?

The research team will explore the relevance of the different TDF domains to oral health advice and support for children and their parents. The 14 research domains are: knowledge, skills, social influence, memory, behaviour regulation, professional/social role and identity, beliefs about capabilities, beliefs about consequences, optimism, intentions, goals, emotion, environmental context and resources and reinforcement.

Parent’s experiences of receiving oral health advice from dental health professionals for their children and factors influencing oral health practices: Focus groups with parents of children aged 0-11 years.- TOPIC GUIDE

| **Explore some background information about the parents and their children in relation to dental attendance?**  **[Icebreaker]** |
| --- |
| First of all, please could you tell me how many children you have?  -*How old are the children?*  -*Do they all attend the dentist?*  -*How often, e.g. regularly for check-ups or when just required, e.g. problem or concern?*  -*Which dentist do they attend?*  *Add few questions to explore taking their children to the dentist… e.g. Are your children happy to go to the dentist? If you have more than one kid, do you bring them all together? Do you go as a family?* |
| **ADVICE – WHAT AND HOW** |
| **Explore the advice that has been provided to the participant in relation to preventive oral health care of their child.** |
| Could you tell me about any advice have you received from the dentist/dental team about looking after your child’s teeth?  ***Explore:***  **What advice did you receive?**  -*Toothbrushing –Supervised or actively brushing your child’s teeth?*  *Frequency, Fluoride toothpaste? Amount of Fluoride? Spitting out the toothpaste, no rinsing after brushing? Type of toothbrush (manual or electric) Did they recommend any specific brand (probe for toothbrush and paste)*  -*Additional Fluoride – Mouthwash, stronger toothpastes (which are prescribed) etc.?*  -*Diet – Amount and Frequency of sugary foods and drinks. Safe snack alternatives? Diet diary or Diet sheet provided to complete and return?*  **How was the advice provided?**  -*Verbal information provided by the dental professional?*  -*TV or Video information at the surgery?*  -*Hands on session, e.g. getting the child to brush in the surgery?*  -*Disclosing solution / purple dye painted onto the teeth or tablet chewed by child?*  -*Leaflets / written information provided? Toothbrushing charts?*  -*Referred to any further guidance for information (games, videos, apps) ?*  -*Any resources used, for example Toothbrushing models?*  *-Did they give you any product sample? If yes, do you remember from which brand?*  **Who provided the advice?**  -*Dentist / Hygienist / Therapist / Dental Nurse*  *What did you think of them providing this advice?/ Were you comfortable with them giving you this advice?*  **How long did this advice session last for?**  *Did you think the advice session was too long/long enough*  *Did you find the session easy to follow? – were there any distractions that made it hard for you to follow?*  **When was it given?**  -*At regular dental check-up appointment?*  -*At separate oral health appointment?*  **Was it followed up at future appointments?**  -*Is advice reinforced at each visit? If so how? How did the dental team reward you/ encourage you and your child if progress was being made (e.g. adopting appropriate behavior)* |
| **Explore preventive treatments provided by the dental teams** |
| **Has your child ever had a high fluoride varnish painted on to their teeth?**  -*A quick procedure of around 2 minutes. Involves painting a yellow/orange varnish / paste on to the teeth, often described as tasting like fruity or like bananas.*  **Who provided this treatment?**  -*Dentist, Hygienist / Therapist / Dental Nurse?*  **When was it carried out?**  - *At check-up appointment or at a different visit to the dentist? If at a different visit, why was this, which member of the dental team provided it, was it in the dental surgery or a different room, how did you feel in this setting?* |
| **IMPACT** |
| **Having received this information from the dental team what effect did it have, why, what did you do,**  **Did you find the advice realistic? What stopped you from taking on board the advice that was given?**  **Explore if the advice has changed or affected the oral health behaviors and home care of the child.**  We would like to find out if the advice you have received has altered your child’s toothbrushing behaviours at home. Can also explore if it has changed wider family behaviour as well  *Do you think that following the advice would make a difference to the oral health of your children (e.g. cavities).*  *How much control do you think you have over your child’s oral health?*  *(If they have multiple children between 0-11) How does your experience with supporting your children’s oral health differ between children or with the same child at different ages?*  *How well do you think you follow the advice the dentist gives for your children?*  *Did you have any problems in following their advice or making sure your child followed their advice?*  *Did the dental team provide clear steps to follow for you and your child, how to measure if you were succeeding, how to manage problems?*  *Did you find it hard to remember the advice that you were given at the dentists? – Were you given any resources to take away with you for refer back to if needed?* |
| **RECOMMENDATIONS FOR BETTER SUPPORT** |
| **Explore what parents think of the advice given and what would their recommendations be for better support**  *-Do you think there are better ways advice could be delivered by the dental team than how you actually get it?*  *-If yes, what would you suggest is the best way to deliver the advice and making sure that they are implemented at home?* |
| **WHAT PRODUCTS AND WHY** |
| **Explore the parents’ views on the oral health resources / products provided.**  We would like to ask your opinions on some dental health products.  - What oral health products do you use for your children? And why?  -*What do you think about these products / materials?*  -*Do you feel that they would / would they help encourage you / your child to want to brush their teeth?*  -*Do you think your children would like them? (may need to explore what it is about the product that would help)*  - *Do you think if your child likes the resources/product this would encourage them to look after their teeth or reduce sugary snacks?*  *Talking about the oral care products (brush, paste, mouthwash…) that you currently use for your kid… do you remember:*   - *How did you find out about the product? Did the kid have any influence in the purchase? Where did you buy them?*   *Probe on brushes vs. paste separately* |

Children’s experiences of receiving oral health advice from dental professionals and oral health behaviours: Focus groups with children aged 7-10 years.- TOPIC GUIDE

| **Explore the children’s experiences of receiving oral health information. [Icebreaker] – language will be adjusted for children of different ages, the text of the topic guide is for the researcher and they will then choose appropriate language to ask the question.** |
| --- |
| **Have you been to the dentist before?**  *-Do you remember the last time when you went?*  *-What did you think of going to the dentist?* |
| **ADVICE: WHAT AND HOW** |
| **Explore the advice that has been provided to the participant in relation to preventive oral health care of their child.** |
| Could you tell me about any advice you have received from the dentist when you visited them?  ***Explore:***  **What did your dentist tell you to do at home to look after your teeth?**  -*Toothbrushing –How often,how long do you brush for? Spitting out the toothpaste, no rinsing after brushing*  *Explore level of parental involvement with brushing*  *Have you ever used an electric toothbrush, if so what type, does it work by batteries or plugs into a charger*  - *Mouthwash etc.?*  -*Diet – Amount and Frequency of sugary foods and drinks, snacks that are good for your teeth, what did they say about what to drink,*  *Did they ask your parents to fill in a diary about what you eat and drink? Did your parents fill it in?*  Are these tips consistent with what your parents tell you to do?  **How was the advice provided?**  -*Did the dentist or nurse tell you what to do at home? And/or did they show you any videos? Did they use any other material / tool? Apps?*  -*Did anybody show you how to brush? Did they use a models (with big teeth) to show how to brush? Did it help you to understand what to do?*  -Did they put any food *dye on your teeth or ask you to chew a tablet? Did it show you where there was still food on your teeth?*  -Did the dental team give you any leaflets, toothbrushing charts or other information/ materials to look after your teeth?  *- did they show you any apps to go on your parent’s phone or tablet which help you to brush your teeth?*  *-Do you remember if you use a brush connected to the app?*  **Can you remember who provided the advice?**  -*Dentist / Hygienist / Therapist / Dental Nurse*  **How long did this advice session last for?**  *Were you listening?*  *Did you find it hard to follow? Did they give you individual steps to follow?*  **When was it given?**  -*At regular dental check-up appointment?*  -*At separate oral health appointment? What it in a different room, did you like going into a room without a dental chair*  **Was it followed up at future appointments?**  - *Do you remember going to the dentist more than one time? If yes, did the dentist ask you if you followed the instructions given to you the first time you visited him/her? And/or were there new things that you were asked to do back home?*  *OR*  *Did the dentist ask you if you have been following his/her advice?*  *-Is advice repeated or new ones provided every time you visit?* |
| **Explore preventive treatments provided by the dental teams** |
| **Have you ever had a varnish painted on to your teeth?**  -*A quick procedure of around 2 minutes. Involves painting a yellow/orange varnish / paste on to your teeth, often described as tasting like fruity or like bananas.*  **Who provided this treatment?**  -*Dentist, Hygienist / Therapist / Dental Nurse?*  **When was it carried out?**  - *At check-up appointment or a separate visit?* |
| **IMPACT** |
| **Having received this information from the dentist what effect did it have, why, what did they do, what are the problems faced in following this advice?**  **Explore if the advice has changed or affected the oral health behaviors and home care of the child.** |
| Have you done anything that your dentist advised you to do at home?  **Do you do anything differently because of something that the dentist said to you?**  *- Did you find it hard to understand what the dentist said?*  *- How well do you think you followed what your dentist asked you to do?*  *- Did you have any problems in following what the dentist asked you to do? Explore topics: Being supervised while brushing, brushing twice a day, regulating diet and changing eating habits if recommended*  *-Did the dental team ask you to use a reminder chart to help you to remember to undertake this behavior?*  *- Did the dentist identify if you had followed their advice (positive recognition of your achievements) and gave different advice next time?* |
| **RECOMMENDATIONS FOR BETTER SUPPORT** |
| **Explore what children think of the advice given and what would their recommendations be for better support**  *-Have you got any ideas how the dentist could make kids like you follow the instructions/advice?* |
| **WHAT PRODUCTS AND WHY** |
| **Explore the child’s views on the oral health resources / products provided.** |
| *What toothbrush and toothpaste products do you use? And why?*  *Who chose the brush for you?*  *We would like to ask your opinions on some dental health products.*  **What do you think about these products / materials?**  *-Would you brush more if you were given one of these?*  *-If you like them, why is that/ what do you like about them?* |
